# PPARα Inhibits Astrocyte Inflammation Activation by Restoring Autophagic Flux after Transient Brain Ischemia

**DOI:** 10.3390/biomedicines11030973

**Published:** 2023-03-21

**Authors:** Doudou Luo, Wenxuan Ye, Ling Chen, Xiaoqian Yuan, Yali Zhang, Caixia Chen, Xin Jin, Yu Zhou

**Affiliations:** 1 Department of Basic Medical Science, School of Medicine, Xiamen University, Xiamen 361102, China; 2 Key Laboratory of Chiral Drugs, Xiamen 361102, China; 3 State Key Laboratory of Cellular Stress Biology, Xiamen University, Xiamen 361102, China

**Keywords:** ischemic stroke, peroxisome proliferator-activated receptor α, astrocyte activation, autophagic flux, lysosome

## Abstract

Astrocyte inflammation activation is an important cause that hinders the recovery of motor function after cerebral ischemia. However, its molecular mechanism has not yet been clearly clarified. The peroxisome proliferator-activated receptor α (PPARα) is a ligand-activated nuclear transcriptional factor. This study aims to further clarify the role of PPARα in astrocyte inflammation activation after cerebral ischemia and to explore the underlying mechanism. Astrocyte activation was induced in an in vivo model by transient middle cerebral artery occlusion (tMCAO) in mice. The in vitro model was induced by an oxygen-glucose deprivation/reoxygenation (OGD/R) in a primary culture of mouse astrocyte. PPARα-deficient mice were used to observe the effects of PPARα on astrocyte activation and autophagic flux. Our results showed that PPARα was mainly expressed in activated astrocytes during the chronic phase of brain ischemia and PPARα dysfunction promoted astrocyte inflammatory activation. After cerebral ischemia, the expressions of LC3-II/I and p62 both increased. Autophagic vesicle accumulation was observed by electron microscopy in astrocytes, and the block of autophagic flux was indicated by an mRFP-GFP-LC3 adenovirus infection assay. A PPARα deficit aggravated the autophagic flux block, while PPARα activation preserved the lysosome function and restored autophagic flux in astrocytes after OGD/R. The autophagic flux blocker bafilomycin A1 and chloroquine antagonized the effect of the PPARα agonist on astrocyte activation inhibition. This study identifies a potentially novel function of PPARα in astrocyte autophagic flux and suggests a therapeutic target for the prevention and treatment of chronic brain ischemic injury.

## 1. Introduction

Ischemic stroke is one of the major causes of death and long-term disability, affecting millions of people worldwide. However, approved effective therapies are very limited. The recombinant tissue plasminogen activator (rt-PA) is the only approved therapy for ischemic stroke, while the narrow therapeutic time window and the reperfusion injury restrict its application for most stroke patients [1]. Therefore, it is very important to develop and to optimize the treatment strategies for ischemic stroke.

Astrocytes, the most abundant cell type in the central nervous system, play multifaceted and vital roles in maintaining the neurophysiological functions of the central nervous system. Astrocytes become activated as a result of inflammatory responses during the progression of pathological changes, such as in brain ischemia [2]. After ischemic stroke, the astrocytes in the boundary zone near the ischemic core are activated. Moderate activated astrocytes promote the healing of lesions, but over-activated astrocytes secrete a large number of inflammatory factors, such as interleukin-1 beta (IL-1β) and tumor necrosis factor alpha (TNF-α), which induce the cascade amplification of neuroinflammatory responses and thus aggravate brain injury [3,4]. Accordingly, the manipulation of astrocyte inflammation activation may help to promote motor function recovery following ischemic stroke [5,6]. However, little is known about the molecular mechanisms underlying the astrocyte inflammatory transition after brain ischemia.

Autophagy is a tightly regulated process in which autophagosomes are formed by the phagocytosis of cytoplasmic proteins or organelles, which then fuse with lysosomes to degrade the contents of the inclusions, so as to realize the metabolic needs and the renewal of certain organelles. Autophagic flux refers to the whole autophagic process, including autophagy induction, phagophore formation, autophagosome maturation, as well as autolysosome formation and degradation [7]. Recently, the involvement of autophagic flux in the regulation of neuroinflammation has drawn substantial scientific interest, and accumulating evidence suggests that the dysregulation of autophagic flux is implicated in the pathogenesis of brain ischemia [8,9].

The peroxisome proliferator activated receptor alpha (PPARα) is a ligand-activated transcriptional factor belonging to the nuclear receptor family [10], which regulates the expression of genes involved in glucose and lipid metabolism, as well as to the inflammatory processes by binding to PPAR response elements in the promoter region of the genes [11]. There are three known PPAR isotypes (PPARα, PPARβ/δ, and PPARγ), and PPARα is the only subtype to colocalize with all cell types in adult mouse and human brain tissues [12]. Certain studies indicate that PPARα is an endogenous neuroprotective factor that predisposes the brain to be more resistant against ischemic stroke [13,14]. Treatments with PPARα agonists, such as fenofibrate, WY-14643, or palmitoylethanolamide exert neuroprotective effects, and these effects are PPARα dependent [15,16,17]. Our previous study indicated that the PPARα agonist oleoylethanolamide (OEA) exerts protective effects against acute cerebral ischemic injury; further, that the protective effect is related to the activation and up-regulation of PPARα in brain tissues [18]. Recently, we have found that OEA inhibits astrocyte activation after brain ischemia by activating PPARα [19]. However, whether and how PPARα mediates the astrocyte inflammatory transition after brain ischemia remains unknown. In addition, PPARα and its agonists were reported to regulate autophagy [20,21]. All these findings prompt us to explore the role of PPARα in the modulation of autophagy during the astrocyte inflammatory activation that occurs after brain ischemia.

Thus, in this study, the effects of PPARα on astrocyte activation and autophagic flux after cerebral ischemia were evaluated. Astrocyte activation in an in vivo model was induced by transient middle cerebral artery occlusion (tMCAO) in mice. Furthermore, the in vitro model was induced by oxygen-glucose deprivation/reoxygenation (OGD/R) in primary cultures of mouse astrocytes. PPARα-deficient mice were used to observe the effect of PPARα on astrocyte activation. In addition, the role of autophagy in the effect of PPARα agonist OEA on astrocyte activation was observed by using an autophagic flux blocker bafilomycin A1 (BAF, in vitro) or chloroquine (CQ, in vivo).

## 2. Materials and Methods

### 2.1. Reagents

The radioimmunoprecipitation assay (RIPA) lysis buffer was purchased from Solarbio (Beijing, China). Dulbecco’s modified Eagle’s medium (DMEM) and fetal bovine serum (FBS) were purchased from HyClone Laboratories, Inc. (HyClone, Logan, UT, USA). Penicillin and streptomycin were purchased from Gibco BRL (Gibco, Carlsbad, CA, USA). All other chemicals and reagents were of analytical grade. BAF, rapamycin (RAPA), and CQ were purchased from MedChem Express (MCE, Monmouth Junction, NJ, USA). The OEA used in the cellular study was purchased from Sigma-Aldrich (St. Louis, MO, USA). The OEA (purity ≥ 98%) used in the animal study was synthesized in our laboratory as previously described [18].

### 2.2. Mice

The PPARα-deficient mice on a 129Sbackground (129S4/SvJae-Pparatm1Gonz/J, stock #003580) were purchased from the Jackson Laboratory (Bar Harbor, ME, USA). The male SV129 mice (8–10 weeks old, 23–25 g) were purchased from Beijing Vital River Laboratory Animal Technology Co., Ltd. (Beijing, China). The postnatal (P1 to P2) SV129 mice or PPARα-deficient mice were used for the primary cortical astrocyte culture. The mice were housed in groups of five per cage, in a temperature and humidity-controlled environment (22 ± 1 °C) with a 12 h light circuit and free access to standard chow and water. Every effort was made to minimize any pain or discomfort, and the minimum number of animals was used.

### 2.3. Transient Focal Cerebral Ischemia Induction

As we have described previously [18,19], the transient middle cerebral artery occlusion (tMCAO) of mice was used for the transient focal cerebral ischemia model. Briefly, the right common carotid artery (CCA), external carotid artery (ECA), and internal carotid artery (ICA) were all exposed when the mice were completely anesthetized with 2% isoflurane (RWD Life Science, Shenzhen, China). A 6-0 nylon monofilament suture was inserted through the CCA into the ICA, then advanced approximately 10 mm to occlude the origin of the middle cerebral artery (MCA). After 30 min of occlusion, the suture was removed to allow reperfusion. The sham group mice were treated with the same procedure, except that the suture was not inserted.

### 2.4. Animal Experimental Protocols and Drug Administration

To observe the effect of the PPARα agonist OEA on autophagy-related protein expressions in the chronic phrase of cerebral ischemia, the mice were randomly divided into four groups: the vehicle-treated sham group, the vehicle-treated tMCAO group, the OEA-treated tMCAO groups, and the CQ-OEA-treated tMCAO groups. OEA was dissolved in Tween-80, and then diluted to a 3% solution with saline. CQ was dissolved with saline. The saline solution with 3% Tween-80 was used as a vehicle control. The OEA (30 mg/kg) or vehicle control was administrated (intraperitoneal injections, i.p.) once a day in a volume of 10 mL/kg from the 3rd day before operation to the 14th day after tMCAO. The CQ (50 mg/kg) was administered (i.p.) every other day in a volume of 10 mL/kg from the 3rd day before operation to the 14th day after tMCAO.

### 2.5. Behavioral Tests

All behavioral tests were conducted prior to and at 1, 3, 5, 7, and 14 days after tMCAO. The animals were pre-trained for 3 days for the rotarod test and balance beam walking test. The order of the assays (i.e., the Bederson scoring tests, balance beam walking test, rotarod test, and the grip strength test) was always the same in order to maintain identical testing conditions for all animals. All tests were carried out according to our previous reports [19,22] and then analyzed by a person who was blind to the experiment.

### 2.6. Magnetic Resonance Imaging (MRI)

The MRI was performed using a 9.4T BioSpec small animal MRI scanner (Bruker, PharmaScan, Ettlingen, Germany), and T2-weighted images were acquired to calculate the hemisphere volume. The mice were anesthetized with 2% isoflurane (RWD Life Science) delivered through a nose cone, whereby the respiratory rhythm and body temperature were carefully monitored by a physiological monitor. The T2-weighted imaging was conducted on the 14th day after tMCAO, using a 2-dimensional fast-spin echo sequence (2500/33 msec of repetition time/echo time, 1 average). For each sequence, 12 axial slices were acquired over the brain, excluding the olfactory bulb, using a field of view of 20 × 20 mm^2^, a slice thickness of 0.5 mm, and a matrix size of 256 × 256. The total scan time was 5 min. The degree of the brain atrophy at striatum or hippocampal level was calculated from the T2-weighted images. The brain atrophy degree at striatum or hippocampal level = (contralateral hemisphere volume-ipsilateral hemisphere volume)/contralateral hemisphere volume). The hemisphere volume was calculated using ImageJ version 1.53 software (NIH, Bethesda, MD, USA).

### 2.7. The Primary Cortical Astrocytes Culture

The primary cortical astrocytes were obtained from SV129 wild type (WT) or PPARα-deficient mice (P1 to P2). Briefly, the mice brains were removed quickly and placed in ice-cold PBS. After the meninges and subcortical tissues were carefully stripped off, the cerebral cortexes were minced and then digested with trypsin-EDTA (Yeasen, Shanghai, China). Subsequently, the cells were planted in poly-L-lysine precoated cell culture flasks containing DMEM (Hyclone, Logan, UT, USA), supplemented with 10% FBS (Hyclone), 1% penicillin/streptomycin, and then cultured in a humidified incubator (37 °C, 5% CO_2_). The cell culture medium was changed 24 h after plating and then, subsequently, every 3 days until reaching confluence, which usually occurred after 7–10 days. The cultures were shaken overnight in order to remove the oligodendrocytes and microglial cells. The purified astrocytes, which were constituted by more than 95% of GFAP-positive cells, were seeded onto plates in serum-containing DMEM. The attached cells were seeded onto new plates for further studies. All experiments were performed on cultures at 21–22 days.

### 2.8. Oxygen Glucose Deprivation and Reoxygenation (OGD/R)

To mimic the astrocyte activation after transient cerebral ischemia in vitro, we established the OGD/R model in the primary culture of mice astrocytes according to a previous report [23]. Briefly, the primary-cultured astrocytes were rinsed twice with phosphate-buffered saline (PBS, pH 7.4), and then refreshed with glucose free DMEM (Gibco). Cells were then placed in a sealed chamber (Billups-Rothenberg, San Diego, CA, USA), loaded with mixed gas containing 95% N_2_, and 5% CO_2_ at 37 °C for 2–6 h. Following the insult, cultures were transferred back to a DMEM containing 10% FBS and reoxygenated in a humidified incubator (37 °C, 5% CO_2_) for an additional 24 h. Cells in the normal control group remained untreated and were incubated in a regular cell culture incubator (37 °C, 5% CO_2_). All astrocytes were harvested 24 h after reoxygenation.

### 2.9. Cellular Experimental Protocols and Drug Treatment

To observe the effect of OGD/R on glial activation, when cells reached 60–70% confluence, the medium was replaced with 2% FBS in a DMEM, and the cells were treated with OGD for 2–6 h, followed by a 24 h reoxygenation. To assay the effect of PPARα on the OGD/R-induced astrocyte activation and autophagic flux after OGD/R, the primary cortical astrocytes were obtained from SV129 WT or from PPARα-deficient mice. To observe the effect of the PPARα agonist OEA on OGD/R-induced astrocyte activation, the cells were pretreated with OEA (50 μM) in a 0.1% DMSO for 12 h before exposure to OGD/R. The astrocytes were treated with the autophagy inducer RAPA (200 nM) or the inhibitor BAF (500 nM) 1 h before OGD exposure in order to determine the role of autophagy in the in vitro model of astrocyte activation. To observe the role of autophagy in the protective effect of the PPARα agonist OEA against OGD/R-induced astrocyte activation, the cells were pretreated with OEA (50 μM) in 0.1% DMSO for 12 h and then the BAF (500 nM) was added 1 h before OGD exposure. The OEA, BAF, and RAPA were dissolved in a DMSO at an initial concentration of 20 mM, 100 mM, and 100 mM, respectively, and further diluted with an appropriate medium. Corresponding amounts of DMSO were added to the control cultures. The final DMSO concentration in the medium did not exceed 0.05% and did not show any effect on the astrocyte cultures.

### 2.10. Protein Preparation and Western Blot Analysis

To extract the protein of the ipsilateral hemisphere brain tissues and cultured cells, the brain tissues and cells were lysed in a RIPA lysis buffer (Applygen Technologies, Beijing, China) with protease (Beyotime, Shanghai, China) and phosphatase inhibitor cocktail (Aidlab, Beijing, China). Then, it was cleared of debris by a centrifugation at 16,000× *g* for 15 min at 4 °C. After measuring protein concentrations with a bicinchoninic acid (BCA) kit (Thermo Fisher Scientific, Waltham, MA, USA), the lysates were boiled with a loading buffer for 10 min. Protein samples (30 μg) from mice brain tissues and astrocyte cultures were separated by 8–12% sodium dodecylsulfate-polyacrylamide gels electrophoresis and transferred to polyvinylidene fluoride membranes (Millipore, Billerica, MA, USA). The membranes were blocked with 5% fat-free milk (Becton, Dickinson and Company (BD),Franklin Lake, NJ, USA) for 1 h and then incubated with one of the following antibodies: rabbit anti-PPARα (1:500, Abcam, Cambridge, MA, USA); rabbit anti-glial fibrillary acidic protein (GFAP) (1:1000, Abcam); sheep anti-neurocan (1:500, R&D systems, Minneapolis, MN, USA); rabbit anti Beclin1 (1:1000, Abcam); rabbit anti-microtubule-associated protein 1 light chain 3 (MAP1LC3/LC3) (1:500, Novus Biologicals, Littleton, CO, USA); rabbit anti-sequestosome 1 (SQSTM1), also known as the ubiquitin-binding protein p62 (p62) (1:4000, Novus Biologicals); rabbit anti IL-1β (1:1000, Proteintech, Wuhan China); rabbit anti TNF-α (1:1000, Proteintech); rat anti lysosomal-associated membrane protein 2 (LAMP2, 1:1000, Abcam); rabbit anti cathepsin D (CTSD, 1:1000, Abcam); rabbit anti-β-actin (1:5000, Proteintech); and rabbit anti glyceraldehyde-3-phosphate dehydrogenase (GAPDH, 1:1000, Proteintech), all at 4 °C overnight. Then, the membranes were incubated with appropriate horseradish peroxidase-conjugated secondary antibodies (1:10,000, Proteintech) for 1 h at room temperature. Finally, the protein bands were revealed by using enhanced chemiluminescence reagents (Millipore Corporation, Bedford, MA, USA), and the images were obtained and analyzed using a Kodak Image Station 4000R (Eastman Kodak Co., Rochester, NY, USA). The optical densities of specific immunopositive bands, namely, those corresponding to PPARα (52 kDa), GFAP (52 kDa), Neurocan (220 kDa), Becline1 (52 kDa); LC3 I/II (15–17 kDa), p62 (62 kDa), IL-1β (12–17 kDa), TNF-α (33–35 kDa), LAMP2 (75 kDa), CTSD (28–46 kDa), GAPDH (36 kDa), and β-actin (42 kDa) were normalized to the GAPDH band or β-actin band in the same sample.

### 2.11. Immunofluorescence Staining

For brain tissues staining, mice were anesthetized with 2% isoflurane (RWD Life Science). Then, the mice were intracardially perfused with ice-cold saline followed by perfusion with 4% paraformaldehyde. The brains were removed and photographed with a digital camera (Nikon D7000, Nikon corporation, Tokyo, Japan). The surface area of ipsilateral (ischemic) or contralateral hemisphere was calculated and compared using ImageJ version 1.53 software (NIH, Bethesda, MD, USA). Degree of brain atrophy = [(contralateral hemisphere area − ipsilateral hemisphere area)/contralateral hemisphere area] × 100%. Then the brains were post-fixed overnight at 4 °C. For cryoprotection, the brains were placed in 15% sucrose in PBS for 6–12 h and 30% sucrose in PBS until the tissue sinks. Next, the brain samples were snap-frozen by immersion in liquid nitrogen and stored at −80 °C until use. The serial 20-μm coronal sections (Bregma 0.5–0.14 mm) were cut by a cryo-microtomy (Leica CM1950, Leica Microsystems, Wetzlar, Germany). Before staining, they were rinsed with PBS, permeabilized by 0.2% Triton-X-100 (Solarbio, Shanghai, China) for 10 min, and blocked with 10% normal goat serum (Boster, Wuhan, China) in PBS for 60 min. Then, they were incubated overnight at 4 °C with the primary antibody, including mouse anti-GFAP (1:200, Cell Signaling Technology Inc. (CST), Danvers, MA, USA), rabbit anti-PPARα (1:200, Abcam), and rat anti-LAMP2 (1:100, Abcam). For the cultured astrocyte staining, cells were fixed with ice cold 4% paraformaldehyde in PBS for 15 min at room temperature. After washing with PBS, the cells were blocked by a 10% normal goat serum blocking solution for 1 h at room temperature and were then incubated overnight at 4 °C with the primary antibodies, including mouse anti-GFAP (1:200, CST), rabbit anti-p62 (1:200, Novus), rabbit anti-PPARα (1:200, Abcam), and rat anti-LAMP2 (1:100, Abcam). Subsequently, the brain sections or cells were incubated with an appropriate Alexa Fluor 594 IgG (1:300; Invitrogen, Thermo Fisher Scientific) or Alexa Fluor 488 IgG (1:300; Abcam) for 2 h. The brain sections or cells were stained with 4, 6-diamidino-2-phenylindole (DAPI, Yeasen) to visualize nuclei. Images were captured by using a confocal microscopy (Olympus FV1000, Tokyo, Japan). The negative controls were carried out by omitting the primary antibody. For the quantifications of the GFAP and LAMP2 fluorescent immunostaining in primary cultured astrocytes, the images were captured by randomly selecting three visual fields, which were pooled from three biological replicates using the same setting parameters and intensity measurements that were performed when using the NIH Image J.

### 2.12. Transmission Electron Microscopy (TEM) Examination

The formation of autophagosomes in astrocytes was examined with TEM. The primary cultured cortical astrocytes were collected by centrifugation (1000 rpm, 5 min), firstly fixed in a 2.5% glutaraldehyde for more than 2 h, and then fixed in a 1% osmium tetroxide. After dehydration in a series of concentrations of ethanol, the samples were embedded in spur resin. Ultrathin sections were cut, mounted onto nickel grids, stained with uranyl acetate and lead citrate, and then examined under TEM H7800 (Hitachi, Tokyo, Japan). Electron micrographs were obtained at a magnification of ×5000 and ×25,000. All experiments and photographs of TEM were supported by the Center of Forecasting and Analysis of Xiamen University, China.

### 2.13. The mRFP–GFP–LC3 Adenoviral Vector Puncta Formation Assay

The mRFP-GFP-LC3 adenoviral particles were purchased from HanBio Technology Co., Ltd. (HanBio, Shanghai, China) and were used to assess the autophagic flux. The primary mouse astrocytes were seeded on the glass cover slips in the 24-well plates and were allowed to reach a 60% confluence at the time of infection. Adenoviral infection was performed according to the instructions of the manufacturer. The astrocytes were incubated in a growth medium with the adenoviruses at a multiplicity of infection (MOI) of 100 for 2 h at 37 °C in the dark. Then, the cells were exposed to OGD/R with or without drug treatment for another 36 h at 37 °C. The cells were washed and fixed with 4% paraformaldehyde. The images were captured using a confocal microscopy (Olympus FV1000, Tokyo, Japan). The numbers of yellow (autophagosome) and free-red dots (autolysosome) were determined by a manual counting fluorescent punctas from at least four randomly selected images with a 60× oil immersion objective per coverslip. At least 40 cells were randomly selected for counting in each group.

### 2.14. Lysosome Labeling with LysoTracker Green DND-26

LysoTracker Green DND-26 is a cell permeable green dye that stains acidic compartments in live cells; as such, it is often used to assay the quantity of lysosome. Briefly, the cells were cultured on cover slips and exposed to OGD/R with or without drug treatment. After, the cells were collected and washed with PBS twice. Then, the cells were incubated with 50 nM of Green DND-26 (CST, Danvers, MA, USA) at 37 °C for 30 min in the dark. After being washed with PBS three times, the cells were immediately observed under a confocal microscopy (Olympus FV1000, Tokyo, Japan).

### 2.15. Statistical Analysis

Values are expressed as the mean ± standard error of the means (SEM). The statistical analysis was performed using one-way analysis of variance (ANOVA) for the differences within treatments followed by Tukey’s post-hoc test or a two-way ANOVA for the differences within time points or treatments, followed by Bonferroni’s post-hoc test (GraphPad Prism version 8.0.1, GraphPad Software Inc., San Diego, CA, USA). Furthermore, a *p* < 0.05 was considered to be statistically significant.

## 3. Results

### 3.1. PPARα Dysfunction Promotes Astrocyte Activation after Transient Brain Ischemia

**Figure 1:**
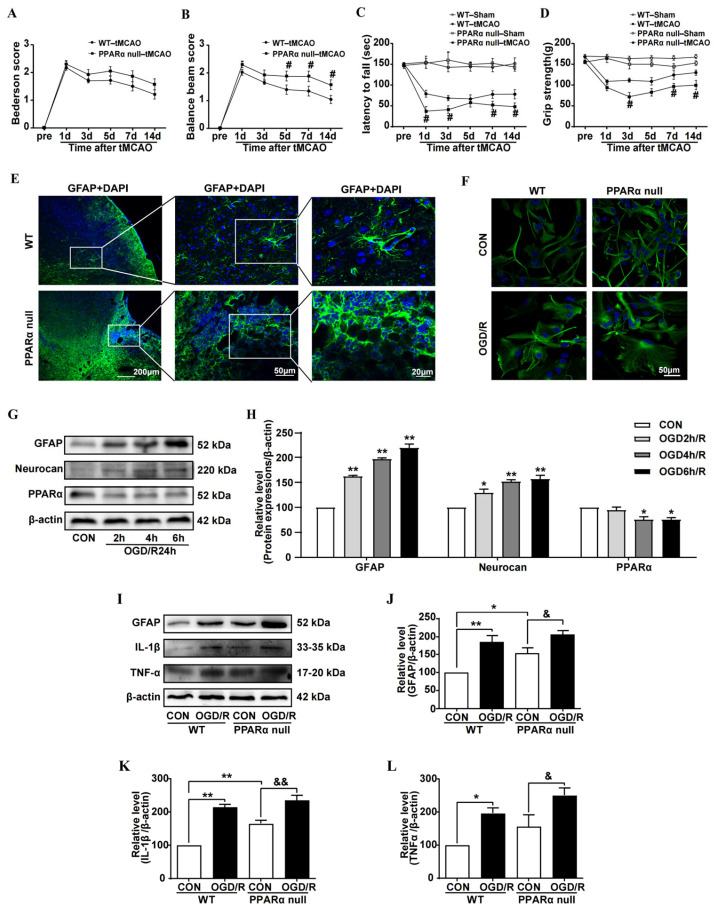
PPARα dysfunction promotes astrocyte activation after transient brain ischemia. The Bederson scoring test (**A**), balance beam walking test (**B**), accelerated rotarod test (**C**), and grip strength test (**D**) were determined over 14 days after tMCAO. The values are expressed as the mean ± SEM; *n* = 3~10; two-way ANOVA for the differences within time points, followed by Bonferroni’s post-hoc test. The # *p* < 0.05 vs. WT tMCAO group. (**E**) The representative photographs of immunofluorescence staining for the GFAP (green) expression in the ischemic boundary zone of brain sections at day 14 after transient brain ischemia. Boxes: areas selected for higher magnification. The representative photographs of immunofluorescence staining for GFAP (green) in the astrocytes from PPARα-deficient mice and its WT control after OGD/R exposure (**F**). The immunoblotting analysis and quantification of GFAP, neurocan, and PPARα expressions in astrocytes after OGD for different times and then reoxygenation for 24 h (**G**,**H**). The immunoblotting analysis and quantification of GFAP, IL-1β, and TNF-α expressions in astrocytes from WT or in PPARα-deficient mice after OGD/R exposure (**I**–**L**). OGD/R means OGD for 6 h and reoxygenation for 24 h. Data are expressed as the means ± SEM. * *p* < 0.05, ** *p* < 0.01 vs. WT normal control; & *p* < 0.05, && *p* < 0.01 vs. PPARα deficit control.

To confirm the role of PPARα in the astrocyte reactivation during the chronic phase after transient brain ischemia, the focal cerebral ischemia injury was induced by tMCAO in PPARα-deficient mice and their WT controls. We first observed the effect of PPARα on neurological function after brain ischemia. Our results show that for the Bederson score, there was almost no significant difference among the groups (*p* > 0.05, Figure 1A). However, both the beam walking ability (Figure 1B) and the motor coordination (Figure 1C) of the PPARα-deficient mice recovered more slowly than the WT controls. In addition, the PPARα-deficient mice showed a slower grip strength recovery when compared with their WT controls (*p* < 0.05, Figure 1D). Consistently, we found that, compared with the WT control mice, the PPARα-deficient mice showed more obvious GFAP expressions in the peri-infarct area 14 days after tMCAO (Figure 1E). All these results indicate that PPARα dysfunction augmented the astrocyte activation and delayed the recovery of motor function after brain ischemia.

To further investigate whether astrocytic PPARα is involved in the modulation of astrocyte activation, OGD/R model was used to mimic the astrocyte activation after brain ischemia in vitro. The results show that after OGD/R, the expressions of specific markers of astrocyte activation and scar formation (GFAP and neurocan) increased significantly (*p* < 0.01, Figure 1F–H), while the PPARα expression in astrocyte decreased more evidently (Figure 1F–H). The expression of the astrocyte activation marker was dramatically increased by OGD for 6 h and reoxygenation for 24 h. Therefore, OGD 6 h/R 24 h was chosen to induce astrocyte activation in the following experiments. Furthermore, we compared the activation degree of the astrocytes that were isolated from the cortex of PPARα-deficient mice and its WT control after OGD/R. Morphologically, OGD/R induced a more evident cell hypertrophy and GFAP expression in the primary culture of the astrocytes that were isolated from the PPARα-deficient mice rather than was observed in their WT control (Figure 1). Consistent with the astrocyte’s phenotype in PPARα-deficient mice, the expressions of astrocyte activation markers (GFAP, IL-1β and TNF-α) in PPARα-deficient astrocytes increased more significantly than its WT control (Figure 1I–L). All these results indicate that PPARα dysfunction promotes astrocyte over-activation after transient brain ischemia.

### 3.2. Autophagic Flux Dysfunction Promotes Astrocyte Activation after Transient Brain Ischemia

**Figure 2:**
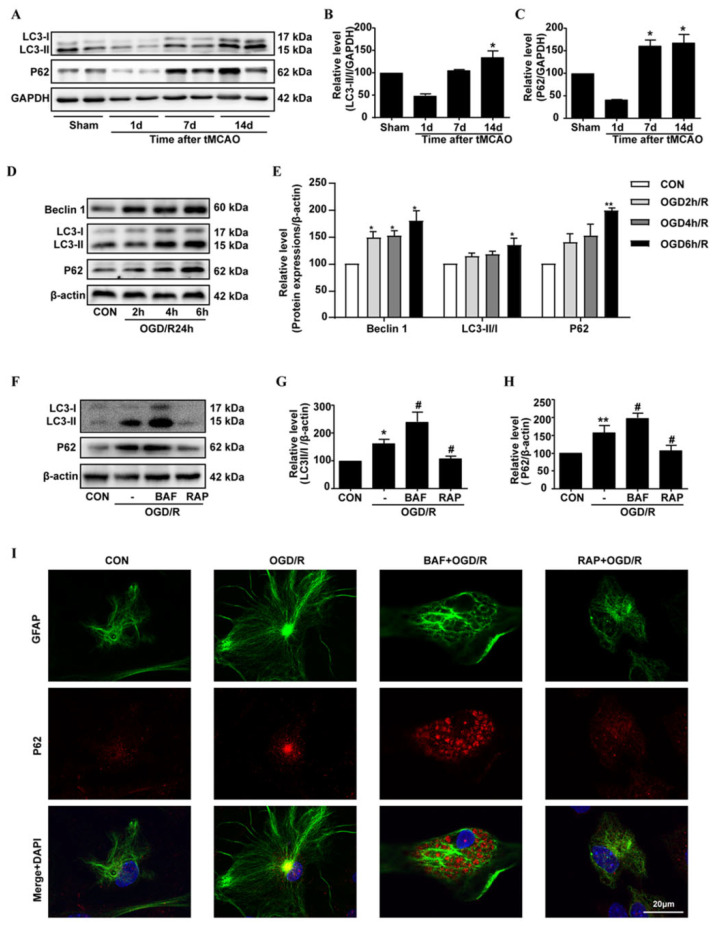
Autophagic flux dysfunction promotes the astrocyte activation after brain ischemia in vivo and in vitro. (**A**–**C**) The immunoblotting analysis and quantification of autophagy markers LC3-II/I and p62 in brain tissues after tMCAO in mice. Data are expressed as the mean ± SEM; *n* = 3; and * *p* < 0.05 vs. sham group. (**D**,**E**) The immunoblotting analysis and quantification of Becline1, p62, and LC3-II/I in astrocytes after exposure to the different times of OGD and then reoxygenation for 24 h. (**F**–**H**) The immunoblotting analysis and quantification of p62 and LC3-II/I in the astrocytes exposed to OGD/R after pretreatment with rapamycin (RAPA, 200 nM) or bafilomycin A1 (BAF, 500 nM). RAPA was used to induce autophagy, and BAF was used to prevent lysosomal acidification and to inhibit the autophagic flux in astrocytes, respectively. (**I**) The representative photographs of the immunofluorescence staining of GFAP (green) and p62 (red) with DAPI (blue) staining in astrocytes were exposed to OGD/R after pretreatment with RAPA or BAF. OGD/R means OGD for 6 h and reoxygenation for 24 h. The data are representative of the three independent experiments and are expressed as the mean ± SEM. * *p* < 0.05, ** *p* < 0.01 vs. normal control group; # *p* < 0.05 vs. OGD/R control group. OGD/R means OGD for 6 h and reoxygenation for 24 h.

In order to investigate the characteristics of autophagic flux after cerebral ischemia, the expressions of the classical autophagy markers, such as LC3 and SQSTM1/p62, in the brain tissues were observed at 1, 7, and 14 days after cerebral ischemia. The results showed that the expressions of LC3-II and p62 both increased after the occurrence of transient cerebral ischemia in mice (Figure 2A–C).

In primary mouse cortical astrocyte culture, the expressions of the autophagy-related protein (Becline1, LC3-II and p62) increased gradually after OGD/R (Figure 2D,E). Treatment with the autophagic flux blocker BAF did not further change the level of p62, thereby suggesting that the autophagic flux was impaired (Figure 2F–H). Particularly, the results of the confocal microscopy assay showed that the BAF promoted the accumulation of p62 and up-regulated GFAP expression (Figure 2I), thus reflecting the block of autophagic flux in reactive astrocytes. On the contrary, the treatment with RAPA, an autophagy inducer, attenuated the accumulation of p62 and inhibited the astrocyte activation (Figure 2I). All these results suggest that a late block of autophagic flux occurred after autophagosome formation, involving either autophagosome/lysosome fusion or lysosomal degradation in the reactive astrocytes that were induced by OGD/R.

### 3.3. PPARα Dysfunction Hinders Autophagic Flux after OGD/R in Primary Mouse Astrocyte Culture

**Figure 3:**
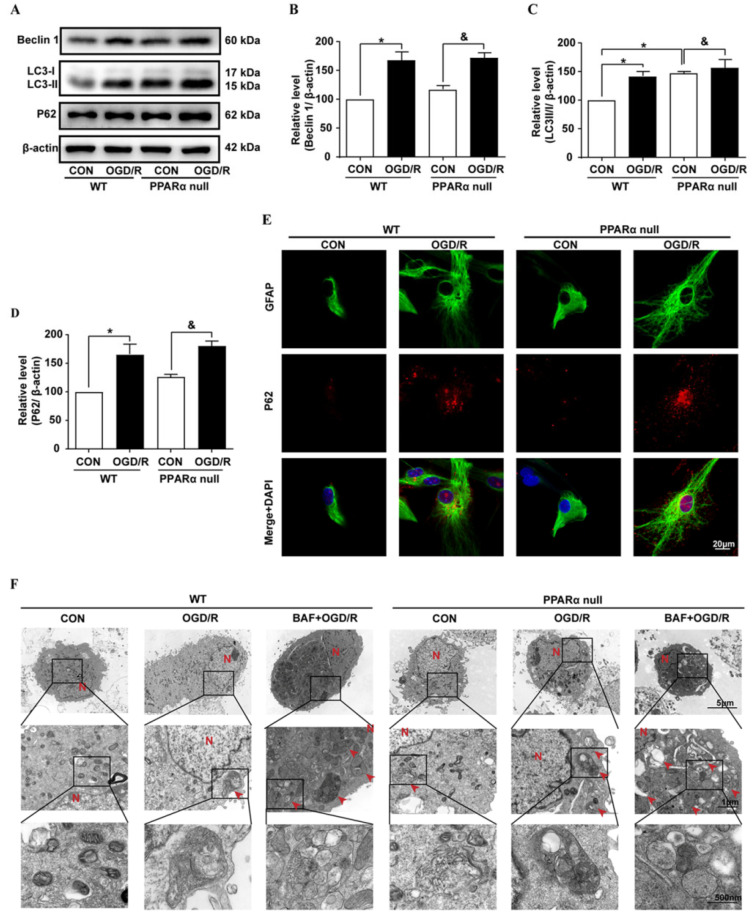
PPARα dysfunction hinders autophagic flux in primary mice astrocyte after exposure to OGD/R. (**A**–**D**) The immunoblotting analysis and quantification of Beclin1, p62, and LC3-II in WT and PPARα-deficient astrocyte exposure to OGD/R. The data are representative of the three independent experiments and are expressed as the mean ± SEM. * *p* < 0.05 vs. WT normal control; & *p* < 0.05 vs. PPARα deficit control. (**E**) The representative photographs of immunofluorescence staining of GFAP (green) and p62 (red) with DAPI (blue) staining in the WT and PPARα-deficient astrocytes after exposure to OGD/R. (**F**) The representative ultrastructural morphology of autophagic vesicles (autophagosome/autolysosome) was observed by TME in WT and PPARα-deficient astrocytes after exposure to OGD/R, whether with BAF treatment or not. Boxes: areas selected for higher magnification. Red arrows indicate autophagic vesicles and N indicates nucleus. OGD/R means OGD for 6 h and reoxygenation for 24 h.

In order to further study whether astrocytic PPARα is involved in regulating autophagy, we isolated primary astrocytes from the cerebral cortex of WT and PPARα-deficient neonatal mice; then, we measured their autophagic flux. We found that PPARα dysfunction increased the LC3-II/LC3-I ratio when compared to its normal WT control (Figure 3A,C), while OGD/R increased both the LC3-II and p62 expressions in the astrocytes of PPARα-deficient mice and their WT control (Figure 3A–D). Particularly, by confocal microscopy assay, we found that p62 expression increased more evidently in PPARα-deficient astrocytes than in the WT control (Figure 3E). According to TME analysis, the astrocytes in the normal control group were normal in morphology, and the autophagic vesicles were rare (Figure 3F). On the contrary, the autophagic vesicles (red arrow heads) were often observed in the OGD/R group and the OGD/R + BAF group. There were more autophagic vesicles in the PPARα-deficient astrocytes than in the WT control after OGD/R exposure (Figure 3F). Taken together, these results suggest that PPARα dysfunction attenuates autophagic flux in the primary culture of mouse astrocytes after OGD/R.

### 3.4. PPARα Agonist OEA Restores Autophagic Flux and Inhibits Astrocyte Activation after Transient Brain Ischemia

**Figure 4:**
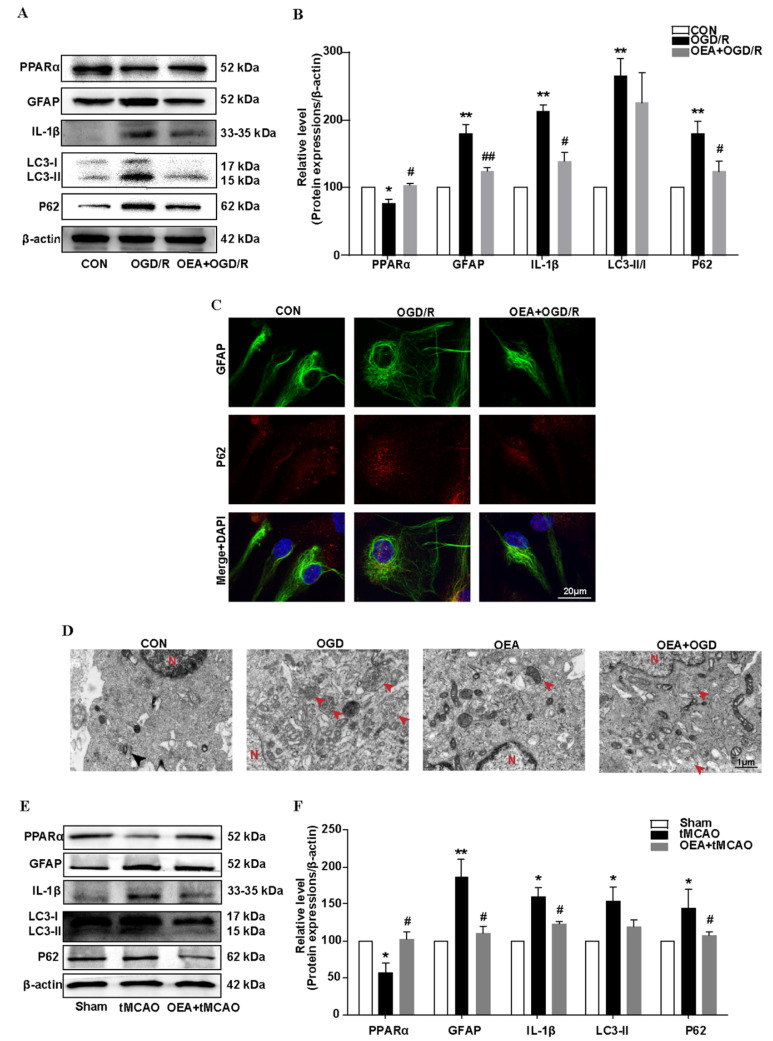
PPARα agonist OEA restores the autophagic flux and inhibits astrocyte activation after brain ischemia in vitro and in vivo. (**A**,**B**) The immunoblotting analysis and quantification of PPARα, GFAP, IL-1β, LC3-II/I, and p62 in vehicle- or OEA-treated astrocytes after exposure to OGD/R. OEA (50 μM) in a 0.1% DMSO was pretreated for 12 h before exposure to OGD/R in the primary astrocytes. Data are representative of the three independent experiments and are expressed as the mean ± SEM. * *p* < 0.05, ** *p* < 0.01 vs. normal control group; # *p* < 0.05, ## *p* < 0.01 vs. OGD/R vehicle group. (**C**) The representative photographs of immunofluorescence staining of GFAP (green) and p62 (red) with Hoechst (blue) staining in vehicle- or OEA-pre-treated astrocytes after exposure to OGD/R. (**D**) The representative ultrastructural morphology of autophagic vesicles (autophagosome/autolysosome) were observed by TME in vehicle- or OEA-pre-treated astrocytes, with or without exposure to OGD/R. Red arrows indicate autophagic vesicles. N indicates nucleus. OGD/R means OGD for 6 h and reoxygenation for 24 h. (**E**,**F**) The immunoblotting analysis and quantifications of PPARα, GFAP, IL-1β, LC3-II/I, and p62 in brain tissues after tMCAO in mice with vehicle- or OEA-pre-treatment. Data are expressed as the mean ± SEM; *n* = 3; * *p* < 0.05, ** *p* < 0.01 vs. sham group; # *p* < 0.05 vs. vehicle-treated tMCAO group.

To determine whether PPARα activation promotes autophagic flux, the PPARα agonist OEA was used. Our results showed that OEA restored the PPARα expression and decreased the expressions of GFAP and IL-1β, which indicated that OEA activated PPARα and inhibited the activation of astrocytes; at the same time, the OEA decreased the expressions of LC3-II and p62, which indicated that OEA restored the autophagic flux (Figure 4A,B). This result was confirmed by the confocal microscopy, which showed that OEA treatment down-regulated the p62 expression in the reactive astrocytes that were induced by OGD/R exposure (Figure 4C). Furthermore, via the TEM analysis of the astrocytes, we also found that OEA decreased the accumulation of large autophagic vesicles in the cytoplasm of astrocytes after OGD/R exposure (Figure 4D). In addition, OEA treatment restored PPARα expression, inhibited astrocyte activation, and promoted autophagic flux in brain tissues at 14 d, after transient brain ischemia occurred in mice (Figure 4E,F). These results suggest that PPARα activation restores autophagic flux and inhibits astrocyte activation after transient brain ischemia.

### 3.5. PPARα Agonist OEA Restores Autophagic Flux by Alleviating Lysosomal Dysfunction in Astrocytes after Brain Ischemia In Vitro and In Vivo

**Figure 5:**
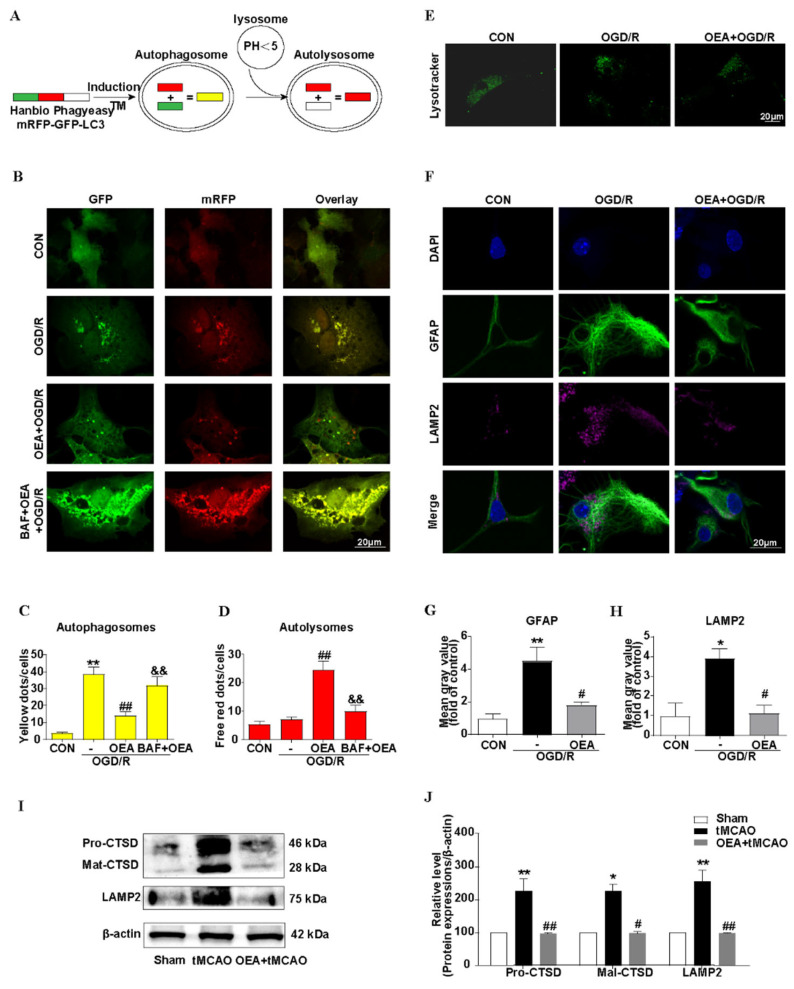
OEA restores autophagic flux by preserving the lysosome function in astrocytes after brain ischemia in vitro and in vivo. (**A**) The schematic diagram of the mRFP-GFP-LC3 plasmid. The primary astrocytes were incubated with mRFP-GFP-LC3 adenoviral particles for 2 h. Then, they were exposed to OGD/R, with or without drug treatment, for another 36 h at 37 °C. (**B**) The representative images of the immunofluorescent astrocytes expressing mRFP-GFP-LC3. Green fluorescence represents GFP, and red fluorescence represents mRFP. (**C**,**D**) The statistics of autophagosomes (yellow dots) and autolysosomes (free-red dots) in each group. The fluorescent punctas were determined by manual counting from at least four randomly selected images with a 60× oil immersion objective per coverslip. At least 40 cells were randomly selected for counting in each group. The OEA (50 μM) was pretreated for 12 h before exposure to OGD/R. The BAF (500 nM) was pretreated 1 h before OGD/R. Data are representative of the three independent experiments and are expressed as the mean ± SEM. ** *p* < 0.01 vs. normal control; ## *p* < 0.01 vs. OGD/R control; && *p* < 0.01 vs. OGD/R+OEA group. (**E**) The representative images showing Lysotracker (Green) staining in primary-cultured astrocytes; (**F**–**H**) The fluorescent immunostaining and quantification of GFAP and LAMP2 in primary-cultured astrocytes (GFAP: green; LAMP2: purple; and Hoechst: blue). Data are representative of the three independent experiments and are expressed as the mean ± SEM. * *p* < 0.05, ** *p* < 0.01 vs. normal control; # *p* < 0.05 vs. OGD/R control; OGD/R represented OGD for 6 h and reoxygenation for 24 h. (**I**,**J**) The immunoblotting analysis and quantification of LAMP2 and CTSD in brain tissues 14 d after tMCAO in mice with vehicle or OEA pre-treatment. Data are expressed as the mean ± SEM; *n* = 3; * *p* < 0.05, ** *p* < 0.01 vs. sham group; # *p* < 0.05, ## *p* < 0.01, vs. vehicle-treated tMCAO group.

To distinguish whether the effect of the PPARα agonist OEA on the accumulation of autophagosomes is due to increase the fusion of autophagosomes with lysosomes or to decrease the autophagosomal formation, the primary mouse astrocytes were transfected with an adenoviral expression vector, mRFP–GFP–LC3, and a double-tagged LC3. The mRFP–GFP–LC3 vector contains an acid-sensitive green fluorescent protein (GFP) and an acid-insensitive monomeric red fluorescent protein (mRFP), which was performed so we could distinguish the autophagosome from autolysosome. In this method, the change from autophagosome (neutral pH) to autolysosome (acidic pH) can be visualized by imaging the quench of the GFP fluorescence, thus leaving only red fluorescence. Therefore, autophagosomes and autolysosomes are labeled with yellow (green and red) or free-red dots, respectively (Figure 5A). Compared with normal control cells, the numbers of the autophagosome were significantly increased and the number of autolysosomes remained unchanged after OGD/R (Figure 5B–D). These results indicate that the fusion of autophagosome to lysosome was blocked and that the autophagic flux was inhibited in the reactive astrocytes that were induced by OGD/R. The PPARα agonist OEA significantly decreased the number of autophagosomes and increased the number of autolysosomes, and these effects were blocked by BAF (Figure 5B–D). All these results suggest that PPARα activation increases the fusion of autophagosomes with lysosomes and then restores the autophagic flux in astrocytes after OGD/R.

As lysosomal function is necessary to support autophagic flux, we further investigate the effect of OEA on lysosome in primary astrocytes after OGD/R. LysoTracker Green DND-26 is a cell permeable green dye that stains acidic compartments in live cells; as such, it is often used to assay the quantity of lysosome. Our results showed that the number of intracellular lysosomes (green, fluorescent dots) decreased during the astrocyte activation that was induced by OGD for 6 h and reoxygenation for 24 h. The OEA restored the number of green, fluorescent dots, indicating that OEA restores the function of lysosomes (Figure 5E). Moreover, cellular LAMP2 accumulation indicates lysosomal dysfunction. Therefore, the effect of OEA on the lysosomes was also examined by observing LAMP2-labeled lysosomes in astrocytes after OGD/R (Figure 5F). Our result indicates that after OGD/R, the volume of LAMP2-labeled lysosome in astrocytes become larger, and that the number of enlarged lysosomes in astrocytes increase (Figure 5H). OEA significantly decreased the number of enlarged LAMP2-labeled lysosomes, thereby suggesting that OEA might help to stabilize the lysosomal membrane (Figure 5F–H). Furthermore, we examined the changes in the expression of LAMP2 and the lysosomal CTSD in brain tissues at 14 d after tMCAO. The results showed that the expressions of the lysosomal markers (LAMP2 and CTSD) significantly increased in the reactive astrocytes, and that OEA restored the level of these two proteins (*p* < 0.01, Figure 5I,J), thus suggesting that OEA might help to restore the function of lysosome.

### 3.6. Chloroquine Antagonizes the Protective Effect of OEA on Chronic Brain Ischemic Injury in Mice

**Figure 6:**
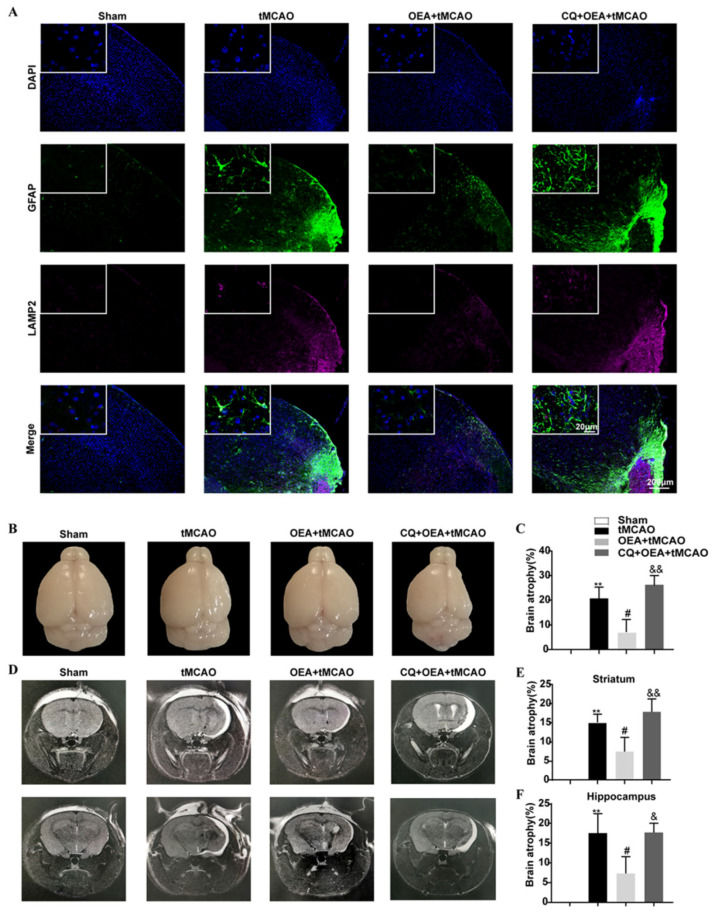
Chloroquine antagonizes the protective effect of OEA on chronic brain ischemic injury in mice. (**A**) The representative photographs of immunofluorescence staining for GFAP (green) and LAMP2 (purple) expressions in the ischemic boundary zone of brain sections after the administration of CQ (50 mg/kg/2 d, i.p. administration from 3 days before surgery to 14 d after tMCAO), with or without the treatment of the PPARα agonist OEA (30 mg/kg/d, i.p. administration from 3 days before surgery to 14 d after tMCAO) at day 14 after tMCAO. The high-magnification images of the reactive astrocytes in the boundary zone are shown in the inserts. (**B**,**C**) The representative photographs and quantitative analysis of the degree of brain atrophy after tMCAO with the administration of OEA, with or without CQ. (**D**–**F**) The representative T2-weighted MRI images and quantitative analysis of brain atrophy degree at striatum and hippocampal levels. Data are expressed as the mean ± SEM, *n* = 3 mice, ** *p* < 0.01 vs. sham group; # *p* < 0.05 vs. vehicle-treated tMCAO group; & *p* < 0.05, && *p* < 0.01 vs. OEA-treated tMCAO group.

We further investigated whether autophagic flux contributed to the protective effect of PPARα against chronic brain ischemic injury. Our results showed that treatment with the PPARα agonist OEA (30 mg/kg/d, i.p.) significantly decreased the expressions of the reactive astrocyte marker GFAP and the lysosome membrane marker LAMP2; these effects were also attenuated by the autophagic flux blocker CQ (Figure 6A). We also found that OEA improved the brain atrophy from 20.9 ± 2.6% to 6.9 ± 3.0% (Figure 6B,C). In contrast, a combined treatment with OEA and CQ (50 mg/kg/2 d, i.p.) did not show any significant protection against the chronic ischemic injury. In addition, the brain atrophy degree was 26.3 ± 2.3% (Figure 6B,C). These results were further confirmed by the results of MRI assay (Figure 6D–F). Thus, blocking autophagic flux in the degradation phase by CQ abolished the protective effects of OEA on astrocyte activation and brain atrophy after brain ischemia. Therefore, all these results indicate that the protection of PPARα activation is mediated by the restoration of autophagic flux after transient brain ischemia.

## 4. Discussion

Astrocyte inflammation activation and glial scar formation are one of the main causes that impend motor function recovery after ischemic strokes [24]. Accordingly, the manipulation of astrocyte inflammation activation may therefore help to facilitate motor function recovery following a stroke. In the present study, we propose a novel mechanism for the PPARα-medicated inhibition of astrocyte inflammation activation by preserving the lysosome function and by restoring autophagic flux through the subsequent lysosomal degradation of autophagic vesicles after transient brain ischemia.

In this study, we found that reduced PPARα expression was shown to be associated with astrocyte inflammation activation after brain ischemia. PPARα-null mice exhibit an aggravated reaction to brain ischemia and delay the recovery of motor function. Although others’ reports have indicated that PPARα-null mice show increased infarct volumes after brain ischemia [25], our study provides new evidence that PPARα null promotes astrocyte inflammation activation and glial scar formation after brain ischemia. Ours and others’ previous studies have shown that the PPARα agonist OEA [19] and palmitoylethanolamide [26] inhibit astrocyte activation and promote motor function recovery after cerebral ischemia. All these results demonstrate the important role of PPARα in inhibiting the neuroinflammation that occurs during the chronic phase of brain ischemia. In addition, they also demonstrate that PPARα could be a potential therapeutic target for ischemic stroke. In actuality, the effect of PPARα on neuroinflammation has been reported in many central nervous system diseases, such as Alzheimer’s Disease and other neurodegenerative disorders [27,28]. As a ligand-activated transcriptional factor, PPARα heterodimerizes with the retinoid X receptor (RXR) in order to regulate the expression of the genes that are involved in inflammatory processes, and which exert anti-inflammatory effects.

In recent years, an increasing number of studies have linked autophagy to inflammatory diseases [29]. Emerging evidence indicates that autophagy plays a regulatory mechanism in controlling the dysregulated and excessive activation of the inflammatory response. Dysfunctional autophagy process and enhanced inflammatory markers are observed in the brain after brain ischemia. In this study, our results demonstrated that astrocyte inflammation activation was associated with LC3-II and p62 accumulation, which strongly indicated that astrocyte activation is associated with an autophagic flux block after brain ischemia. This result is consistent with a previous report, whereby both LC3-II and p62 increased in human postmortem tissue after stroke [30]. In addition, in the primary mouse astrocyte culture, we found that the autophagic flux blocker BAF caused the accumulation of p62 and promoted astrocyte activation. The role of autophagy after brain ischemia likely depends upon the degree of brain injury and the overall autophagic burden. Interventions that stimulate autophagic flux, thereby reducing autophagosome abundance and enhancing autophagosome clearance, may afford neuroprotection in various experimental models [31,32]. In contrast, impaired autophagic flux contributes to astrocyte activation after many central nervous system diseases, such as Alzheimer’s disease [33] and traumatic brain injury [34]. Thus, restoring the autophagic flux in astrocytes may be a novel therapeutic strategy for ischemic stroke.

In this study, we found that the regulation of autophagic flux via PPARα signaling is a novel way to inhibit the astrocyte inflammation activation after ischemic stroke. We found that a PPARα deficit blocked autophagic flux in primary mouse astrocytes after OGD/R exposure. Conversely, the PPARα agonist OEA promoted autophagic flux and inhibited astrocyte inflammation activation after transient brain ischemia. Our findings suggest that the effect of PPARα on the autophagy-inflammatory pathway may provide a new direction for the treatment of ischemic stroke. Furthermore, more and more studies indicate the effect of PPARα on autophagy modulation in various diseases. Certain studies also indicate that PPARα activation mediates autophagy and reduces cognitive decline in a murine model of Alzheimer disease [35]. PPARα activation attenuates the inflammatory response and protects the liver from acute failure by promoting the autophagy pathway [36]. The PPARα agonist OEA alleviates hyperlipidemia-mediated vascular calcification via triggering autophagy by activating PPARα [37]. All these results establish PPARα as potential targets for a therapeutic modulation of the autophagic flux, which could impact the pathogenesis of a wide range of human diseases.

**Figure 7:**
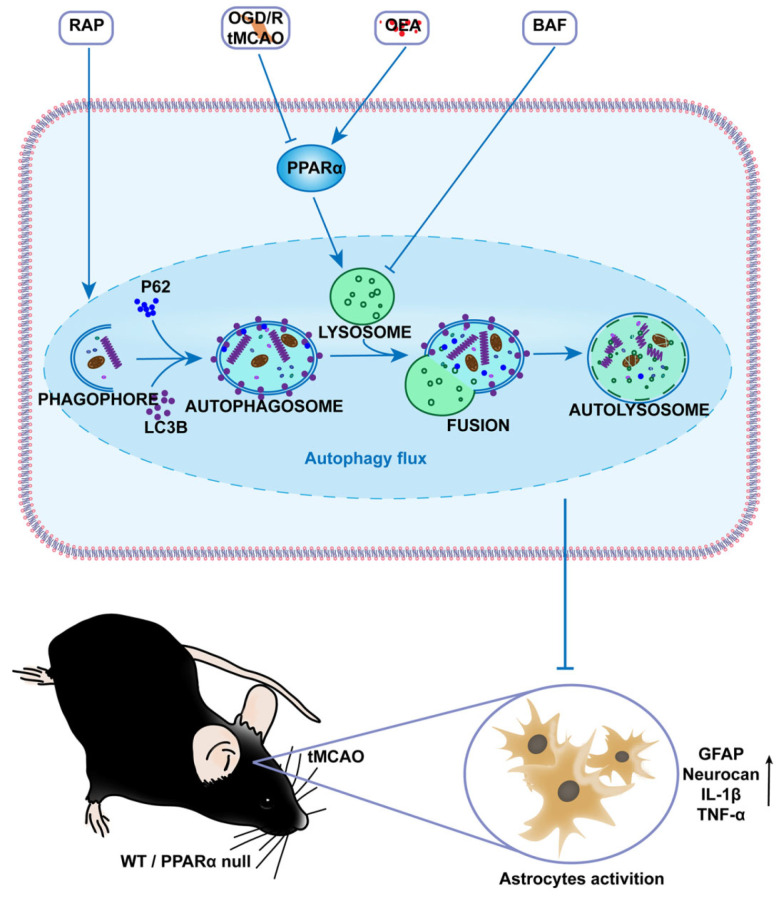
Schematic diagram illustrating the mechanism of the PPARα-medicated inhibition of astrocyte inflammation activation after brain ischemia. PPARα activation preserves the lysosome function and restores autophagic flux through the subsequent lysosomal degradation of autophagic vesicles after transient brain ischemia. Upwards arrow represents upregulation of the substances.

Although the precise mechanism of the effect of PPARα on astrocyte autophagic flux after brain ischemia is not well understood, lysosomes in astrocytes may play an important role. Many studies indicate that the activation of PPARα stimulates lysosomal biogenesis in brain cells [38] via the transcriptional up-regulation of the transcription factor EB (TFEB) [39]. In our research, by using an adenoviral expression vector, mRFP–GFP–LC3, we found that the autophagosome- lysosomes fusion was blocked, and was then attenuated to the autophagic flux in activating astrocytes that were induced by OGD/R. The PPARα agonist OEA significantly decreased the number of autophagosomes and increased the number of autolysosomes, and these effects were blocked by BAF. BAF is a specific inhibitor of the vacuolar-type H^+^-translocating ATPase (V-ATPase) inhibitor and therefore affects the acidification of lysosomes, which then impairs the lysosome function [40]. Lysosomes are specialized cellular organelles, which are critical for the maturation stage of autophagy. Moreover, the fusion of autophagosomes with lysosomes to form the autolysosome is necessary for autophagic flux [41,42]. By the LysoTracker Green staining, our results indicated that, after OGD/R, the acidification of lysosomes in the astrocytes decreased, and the PPARα agonist OEA restored the function of the lysosomes. LAMP2 contributes to the fusion process of autophagosomes and lysosomes; further, LAMP2 accumulation also indicates lysosomal dysfunction [43]. Our results indicated that OEA inhibited LAMP2 accumulation. In addition, the protective effect of OEA on brain atrophy after brain ischemia was blocked by CQ, which alkalinizes the lumen of the lysosome and then inhibits its pH-sensitive functions [44]. These results clearly highlight that PPARα activation preserves the lysosome function, restores autophagic flux, and then inhibits the activation of astrocytes after brain ischemia (Figure 7). However, the precise mechanism of PPARα on the protective effect of lysosomes should be studied further.

Another interesting finding in our study is that we observed increased nuclear p62 accumulation and decreased cytoplasmic p62 in activated astrocytes after OGD/R. PPARα null promotes p62 nuclear accumulation in astrocytes. Conversely, the PPARα activator OEA restored the distribution of p62 in astrocytes. Furthermore, p62 is a multifunctional stress-inducible scaffold protein that is involved in diverse cellular processes. Cytosolic p62 plays an important role in maintaining protein homeostasis, which is mainly defined as a cargo receptor for autophagy and is a process that allows the degradation of detrimental and unnecessary components through the lysosome [45]. Besides this role, p62 could also interact with many other partners. For example, in its ability to interact with multiple binding partners, such as the nuclear factor erythroid 2- related factor 2(Nrf2), the mechanistic target of rapamycin complex 1 (mTORC1), and the nuclear factor kappa-light-chain-enhancer of activated B cells (NF-κB) signaling pathways, which link p62 to the oxidative defense system, nutrient sensing, and inflammation, respectively [46,47]. However, the function of nuclear p62 remains relatively unknown and needs further investigation.

Definitely, there are still certain limitations in our study. Firstly, we found that a PPARα deficit promotes astrocyte inflammation activation and retards motor function recovery after brain ischemia. However, the effect of PPARα on other cells after brain ischemia, such as neurons and endothelial cells, remains relatively unknown. In future studies, conditional PPARα knockout mice targeting astrocytes, neurons, and endothelial cells should be used to provide a more comprehensive understanding of the effect of PPARα on brain ischemic injury. Secondly, we found that PPARα preserves the lysosome function and restores the autophagic flux. However, more detail on the molecular mechanisms underlying the effect of PPARα on lysosome function is required. Third, in this study, we only observe the effect of OEA on chronic brain ischemic injury. The effects of certain other PPARα agonists, such as Wy14,643 and fenofibrate, on astrocyte inflammation activation and chronic brain ischemic injury could also be studied in the future. These results will open new possibilities for PPARα agonists in medicine against brain ischemia.

In summary, this study identifies the role of PPARα in inhibiting the inflammation activation of astrocytes during the chronic phase of cerebral ischemia, and provides a new insight into the importance of PPARα in regulating neuroinflammation, partially through an autophagic flux regulation. Further preclinical studies on PPARα agonists are warranted for the development of a clinically applicable therapeutic strategy against chronic brain ischemic injury.

## Data Availability

The data presented in this study are available upon request from the corresponding authors.
